# Endothelial colony-forming cells reduced the lung injury induced by cardiopulmonary bypass in rats

**DOI:** 10.1186/s13287-020-01722-7

**Published:** 2020-06-25

**Authors:** Haibin Sun, Xiaoqing Zhao, Qihang Tai, Guangxiao Xu, Yingnan Ju, Wei Gao

**Affiliations:** 1 Department of Anesthesiology, The Second Affiliated Hospital of Harbin Medical University, Harbin, China; 2 Department of ICU, Tumor Hospital of Harbin Medical University, Harbin, China

**Keywords:** Endothelial colony-forming cells, Cardiopulmonary bypass, Inflammation, Lung injury

## Abstract

**Background:**

Cardiopulmonary bypass (CPB) results in severe lung injury via inflammation and endothelial injury. The aim of this study was to evaluate the effect of endothelial colony-forming cells (ECFCs) on lung injury in rats subjected to CPB.

**Methods:**

Thirty-two rats were randomized into the sham, CPB, CPB/ECFC and CPB/ECFC/L-NIO groups. The rats in the sham group received anaesthesia, and the rats in the other groups received CPB. The rats also received PBS, ECFCs and L-NIO-pre-treated ECFCs. After 24 h of CPB, pulmonary capillary permeability, including the PaO_2_/FiO_2_ ratio, protein levels in bronchoalveolar lavage fluid (BALF) and lung tissue wet/dry weight were evaluated. The cell numbers and cytokines in BALF and peripheral blood were tested. Endothelial injury, lung histological injury and apoptosis were assessed. The oxidative stress response and apoptosis-related proteins were analysed.

**Results:**

After CPB, all the data deteriorated compared with those obtained in the S group (sham vs CPB vs CPB/ECFC vs CPB/ECFC/L-NIO: histological score 1.62 ± 0.51 vs 5.37 ± 0.91 vs 3.37 ± 0.89 vs 4.37 ± 0.74; PaO_2_/FiO_2_ 389 ± 12 vs 233 ± 36 vs 338 ± 28 vs 287 ± 30; wet/dry weight 3.11 ± 0.32 vs 6.71 ± 0.73 vs 4.66 ± 0.55 vs 5.52 ± 0.57; protein levels in BALF: 134 ± 22 vs 442 ± 99 vs 225 ± 41 vs 337 ± 53, all *P* < 0.05). Compared to the CPB treatment, ECFCs significantly improved pulmonary capillary permeability and PaO_2_/FiO_2_. Similarly, ECFCs also decreased the inflammatory cell number and pro-inflammatory factors in BALF and peripheral blood, as well as the oxidative stress response in the lung tissue. ECFCs reduced the lung histological injury score and apoptosis and regulated apoptosis-related proteins in the lung tissue. Compared with the CPB/ECFC group, all the indicators were partly reversed by the L-NIO.

**Conclusions:**

ECFCs significantly reduced lung injury induced by inflammation after CPB.

## Background

Postoperative lung injury after cardiopulmonary bypass (CPB) is a rare but severe complication that prolongs the duration of mechanical ventilation and the hospital stay and even increases mortality [1, 2]. The morbidity of post-CPB lung injury is 0.4% to 0.6%, but the mortality of these patients who experience lung injury is approximately 15% to 41.5% [3]. CPB-induced lung injury is associated with systemic inflammation induced by the introduction of blood elements to artificial circuits [2] and lung ischaemia/reperfusion injury [4], and activated inflammatory cells ultimately contribute to alveolar inflammation [5]. Currently, there is no ideal strategy for application in clinical work [6].

As the outgrowth of endothelial progenitor cells, endothelial colony-forming cells (ECFCs) have high proliferative potency [7] and anti-inflammatory effects [8]. ECFCs reduced ischaemic injury and ventilator-induced lung injury via their high proliferative capacity and anti-inflammatory effects [9–11]. Considering the key role of inflammation in lung injury induced by CPB, we hypothesized that ECFCs can ameliorate lung injury after CPB. In this study, we established a rat CPB model to observe the effect of ECFCs on CPB-related lung injury. Previous studies have shown that NO produced by eNOS controls vascular remodelling and angiogenesis. The AKT/eNOS pathway has been suggested to play a pivotal role in bioactivation of endothelial progenitor cells, such as their mobilization, differentiation, homing and angiogenic properties [12, 13]. Moreover, in our previous study, we found that eNOS inhibitor (L-NIO) decreases the recruitment of endothelial progenitor cells in transplanted lung [14]. Therefore, in this study, we postulated that eNOS plays a pivotal role in the bioactivation of ECFCs. To investigate this hypothesis, we pre-treated ECFCs with L-NIO to investigate the possible mechanism of ECFCs on lung injury after CPB.

## Methods

### Animals

This study was approved by the Animal Care and Use Committee of the Harbin Medical University (SYOW2019-157). All animal experiments performed in this study conformed to the National Institutes of Health Guide for the Care and Use of Laboratory Animals. Male Sprague-Dawley rats (approximately 400–450 g) were purchased from the animal centre of the Second Affiliated Hospital of Harbin Medical University.

### In vitro experiments

#### Isolation of ECFCs

We isolated and cultured ECFCs from Sprague-Dawley rats according to a previous study [10]. First, we collected peripheral blood and isolated mononuclear cells using density-gradient centrifugation with Ficoll-Plaque Plus (Amersham Pharmacia Biotech, Uppsala, Sweden). The mononuclear cells were cultured with endothelial growth medium-2 (containing 2% foetal bovine serum) (Lonza, Basel, Switzerland). The mononuclear cells were cultured in six-well plates, which were coated with human fibronectin at 37 °C for 21 days. After 21 days, the adherent cells were harvested for further characterization. The ECFCs in this study are the second generation.

#### Characterization of ECFCs

The cells were identified according to the results of our previous study [10]. Approximately 4 × 10^4^ cells/well were incubated with fluorescein isothiocyanate (FITC)-conjugated *Ulex europaeus* agglutinin-1 (50 μg/ml) (UEA-1, Sigma-Aldrich, Saint Louis, USA) and DiI-acetyl-low-density lipoprotein (LDL) (30 μg/ml) (Invitrogen, Carlsbad, USA). After incubation with UEA and LDL, the mononuclear cells were examined using fluorescence confocal microscopy. The mononuclear cells with dual-positive staining for UEA-1 and acetyl-LDL were defined as endothelial progenitor cells. The cells were also identified with staining for vascular endothelial growth factor receptor (VEGFR) 2 (Abcam, Cambridge, UK) and CD34 (Santa Cruz Biotechnology, Santa Cruz, USA) using a fluorescence microscope. The mononuclear cells with double-positive staining for VEGFR-2 and CD-34 were also identified as endothelial progenitor cells. Based on these results, the cells were further analysed with FITC-labelled CD14 and PE-labelled CD45 antibodies using flow cytometry. The endothelial progenitor cells with double-negative staining of CD14 and CD45 were identified as ECFCs [15]. For analysis of the mechanism of ECFCs in lung injury, ECFCs were preincubated with N5-(1-iminoethyl)-l-ornithine (L-NIO, 10 μM, Santa Cruz Biotechnology) for 1 h to observe the function of the ECFCs [14].

#### Cell proliferation assay

The cellular viability and proliferation of ECFCs were judged by the 3-(4,5-dimethylthiazol-2-yl)-2,5-diphenyltetrazolium bromide (MTT) assay. Approximately 6 × 10^3^ ECFCs/well (pre-treated with or without L-NIO) were plated in 96-well plates in EGM-2 medium. After incubation for 24 h, all the ECFCs were incubated in EBM-2 medium and 1% FBS without serum for 12 h. The ECFCs were then cultured in EBM-2, 1% FBS and VEGF (100 ng/mL). After 24 h, MTT (5 ng/ml) was added, and the ECFCs were incubated for 4 h at 37 °C. Dimethylsulfoxide (150 μl) was injected into the plates, and the plates were further incubated for 10 min. The absorbance of the cells was investigated using Multiskan EX (Thermo, Finland) at 540 nm.

In this study, we also detected the tube formation activity of ECFCs according to a commercial assay kit (Abcam, Toronto, Canada). Briefly, Matrigel (50 μl) was added to each well of a 96-well plate and then incubated at 37 °C with 5% CO_2_ for 30 min to solidify the Matrigel. Next, ECFC cells (10^4^ cells/well) pre-treated with or without L-NIO were seeded onto a 96-well plate with Matrigel. After 12 h, the ECFCs were washed with PBS and the tube network was imaged using an IX51 research microscope. Meanwhile, ECFC cell tube formation was quantitatively measured using ImageJ software.

#### The expression of eNOS in ECFCs

The ECFCs treated with or without L-NIO were harvested, and the total protein of the ECFCs was extracted. eNOS protein expression in ECFCs was detected by Western blotting to investigate the effect of L-NIO on the expression of ECFCs.

### In vivo experiments

#### Rat CPB model

Thirty-two male Sprague-Dawley rats (400–450 g), obtained from the animal centre of the Second Affiliated Hospital Harbin Medical University, were randomized into 4 groups: the sham, CPB, ECFC and ECFC/L groups. The rats in the sham group received only anaesthesia and tracheal intubation. The cells were pre-treated with L-NIO. Briefly, the rats were anaesthetized with 3% pentobarbital sodium (30 mg/kg) intraperitoneally. After anaesthesia, all rats were intubated and ventilated (Model 683, Harvard Apparatus, Boston, USA). The respiratory parameters were a tidal volume (Vt) of 10 ml/kg and a respiratory rate (RR) of 50 breaths/min. The fraction of inspired oxygen (FiO_2_) and positive end-expiratory pressure (PEEP) was set at 50% and 2 cmH_2_O, respectively, and the inspiratory expiratory ratio was 1:1.

After heparinization (500 IU/kg heparin), 18 G and 16 G catheters were inserted into the right carotid artery and right femoral vein, respectively, to inflow and outflow the blood. Moreover, a 22-G catheter was inserted into the right femoral artery to monitor and analyse the blood sample. The CPB circuit was constructed with a 20-ml venous reservoir, roller pump (Cole Parmer instrument company, Chicago, USA) and membrane oxygenator (MeicroPort, Dongguan, Guangdong, China). Before CPB, the circuit was primed with 0.2 ml of heparin, 11 ml of hydroxyethyl starch solution and 0.5 ml of 7% sodium bicarbonate solution [16]. During the experiment, the rectal temperature was monitored and maintained within 36–38 °C by a heat blanket. The flow rate was gradually adjusted to 100 ml/kg body weight/min and maintained for 60 min [17]. During CPB, the mean arterial pressure was maintained within the range of 60 to 80 mmHg using the continuous injection of adrenaline. The anaesthesia was maintained with 3% pentobarbital sodium (10 mg/kg) and rocuronium (0.6 mg/kg) for a 1-h interval. After 60 min of CPB, the outflow cannula was withdrawn, and the right femoral vein was ligated. The remaining priming solution was continuously infused when the haemodynamics were stable, and the inflow catheter was withdrawn. Immediately after withdrawal of the catheter, the rats in the sham and CPB groups were intravenously injected with 1 ml of PBS, and the rats in the ECFC and ECFC/L groups were intravenously injected with ECFCs or ECFCs pre-treated with L-NIO (approximately 10^6^ cells in 1 ml of PBS) [14] via the femoral vein for bolus injection. To prevent infection, 2000 U/kg penicillin was administered, and incisions were sutured. All rats were extubated when they recovered spontaneous breathing. All rats were sacrificed with an overdose of anaesthetics at 24 h after ventilation [18]. In this study, we enrolled 10, 9 and 9 rats in the CPB, ECFC and ECFC/L groups, respectively, to achieve 8 rats in each group.

#### ECFCs and alveolar-capillary permeability

The arterial blood was analysed pre-CPB and at 24 h after CPB using a Bayer Rapidlab 348 (Bayer Diagnostics, Germany). The PaO_2_/FiO_2_ ratio was calculated to evaluate the effect of ECFCs on the lung gas exchange function.

Moreover, part of the lung tissue from the right upper lung lobes was harvested. The lung tissues were weighed and dried at 60 °C for 48 h and then weighed again. The wet/dry weight (W/D) was calculated to observe the effect of ECFCs on alveolar-capillary permeability. Moreover, the protein levels in BALF were also tested.

#### Histopathologic injury evaluation

The lung tissue from the right lower lobe was collected to estimate histological changes. Lung tissue fixed with 4% paraformaldehyde was embedded in paraffin. The lung tissue was cut into 4-μm sections and stained with haematoxylin and eosin. Two independent pathologists were blinded and employed to evaluate lung histological injury with light microscopy.

The lung injury analysis was performed by two pathologists who did not participate in this study. Briefly, the pathology indexes included alveolar congestion, lung oedema, haemorrhage, infiltration of neutrophils into the airspace/vessel wall and alveolar wall thickness [10]. The scoring was as follows: lung haemorrhage (0 = no haemorrhage, 1 = mild haemorrhage, 2 = severe haemorrhage), pulmonary interstitial oedema (0 = no oedema, 1 = mild oedema and 2 = severe oedema), pneumocyte hyperplasia (0 = no alveolar wall thickening, 1 = mild alveolar wall thickening, 2 = severe alveolar wall thickening and 3 = severe alveolar wall thickening with > 50% pulmonary consolidation) and infiltration of inflammatory cells (0 = no inflammatory cell infiltration, 1 = mild inflammatory cell infiltration, 2 = moderate and extensive inflammatory cell infiltration and 3 = severe inflammatory cell infiltration). The lung injury score was I (0–2 score), II (3–6 score), III (7–8 score) or IV (9–10 score).

#### ECFCs and local and systemic inflammation

The right bronchi were blocked using an artery clamp. Sterile saline (15 ml/kg) at 4 °C was injected into the left lung via the left bronchi and was withdrawn 5 times. After 5 withdrawals, the bronchoalveolar lavage fluid (BALF) was collected and centrifuged at 4 °C and 1000*g* for 15 min, and then the supernatant was collected and stored at − 80 °C. The peripheral blood was collected pre-CPB and at 24 h after CPB. The blood was centrifuged at 4 °C and 1500*g* for 10 min, and the serum was collected and stored at − 80 °C. The cytokines TNF-α, IL-1β, IL-6 and IL-10 were detected in the BALF and serum with the corresponding ELISA kits (Wuhan Boster Bio-Engineering Limited Company, Wuhan, China).

Moreover, the number of neutrophils and the levels of elastase in BALF were also detected.

#### ECFCs and oxidative stress response

The lung tissue was isolated and prepared to detect the concentrations of cyclic guanosine monophosphate (cGMP) (Cayman Chemical, Michigan, USA) and superoxide anion and the activity of superoxide dismutation (SOD) using the commercial kits (Solarbio, Beijing, China). Moreover, the expression levels of iNOS, eNOS and phosphorylated eNOS were also detected in the lung tissue using Western blot.

#### Tracking of ECFCs in lung tissue

To observe the distribution of ECFCs in lung tissue, approximately 1 × 10^6^ ECFCs (with or without pre-treated L-NIO) labelled with acetyl-LDL were injected into rats of the ECFC and ECFC/L groups. After 24 h of CPB, the lung tissue was harvested, and ECFC tracking was performed by fluorescence microscopy. A slice of lung tissue was prepared according to the histological analysis method. The pulmonary tissue slices were *deparaffinized* and stained with 4,6-diamidino-2-phenylindole (DAPI) to stain the cell nuclei. The ECFCs in lung tissues were visualized by fluorescence confocal microscopy at a wavelength of 555 nm (acetyl-LDL).

#### Apoptosis assay

Apoptosis in the lung tissue was investigated by TUNEL staining with an Apoptosis Assay kit (Roche, Mannheim, Germany). Briefly, the lung tissue slices were immersed in proteinase K at 37 °C for 30 min. The slices were washed twice with PBS. Then, the slides were incubated in the TUNEL reaction mixture (TdT and fluorochrome-conjugated dUTP) for 60 min in a dark chamber at 37 °C. After washing twice, the slides were further incubated with 1 μg/ml 4,6-diamidino-2-phenylindole for 30 min.

The slides were covered with 0.3% H_2_O_2_ to inhibit endogenous peroxidase activity, incubated with extra-avidin peroxidase and then immersed in diaminobenzidine solution. The nuclei that were stained brown were judged as apoptotic cells. In this study, apoptosis of the endothelium and epithelium was identified by two pathologists who analysed histological injury. The apoptosis index was calculated by the ratio of positive apoptotic cells to total cells in a random field from all slides.

#### Western blot

First, the protein was extracted, and the protein levels were calculated with the Bradford assay. An equivalent protein volume of every sample was injected into the gel. After electrophoresis, the protein was transferred onto a polyvinylidene fluoride membrane. The membrane was blocked with 5% milk for 30 min and incubated with primary antibody [Bax, Bcl-2, cleaved caspase-3, phosphorylated myosin light chain (p-MLC) (Sigma Aldrich, St. Louis, Missouri, USA) and phosphorylated NF-κB (p-NF-κB) (Santa Cruz Biotechnology, CA, USA)] overnight at 4 °C. After washing 3 times with PBS, the membrane was incubated with secondary antibody (Santa Cruz Biotechnology). After reaction with horseradish peroxidase, the bands were visualized with enhanced chemiluminescence.

#### Statistical analysis

The primary outcome of this study is the PaO_2_/FiO_2_ after 24 h of CPB. In the preliminary study of 5 rats, the PaO_2_/FiO_2_ at 24 h post-CPB was 240 ± 33. The sample size was calculated using PASS 11. Eight rats were needed in each group to detect an increase of 30 in the PaO_2_/FiO_2_ with a power of 0.9 and an *α* of 0.05. All the data were normally distributed and are presented as the mean (SD). The data were analysed by one-way analysis of variance and an unpaired *t* test. All data were analysed using IBM SPSS Statistics 19.0 (SPSS, Chicago, IL, USA). A two-tailed *P* value of < 0.05 was considered statistically significant.

## Results

### Characterization of ECFCs

**Fig. 1:**
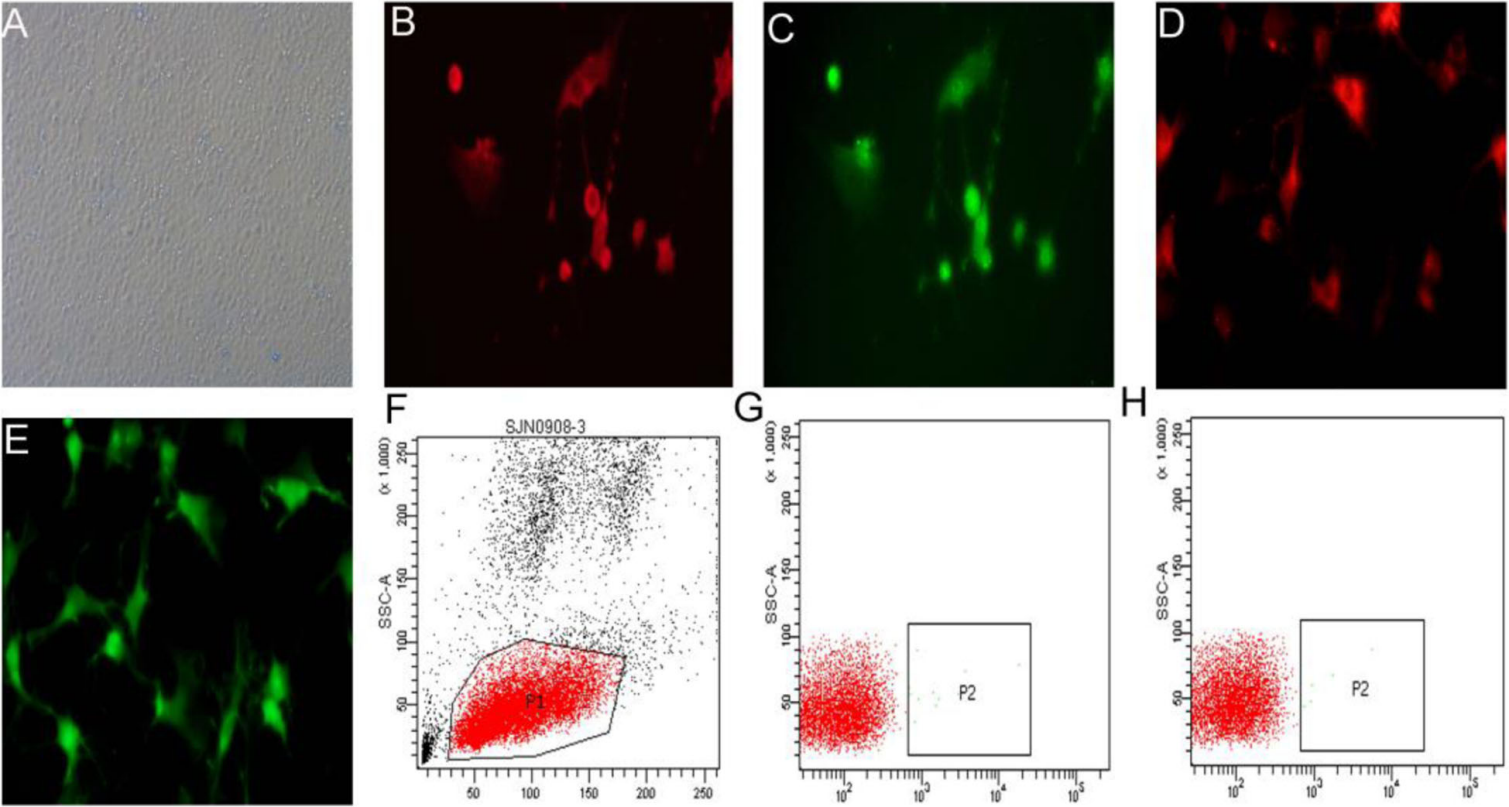
Characterization of ECFCs in vitro. Mononuclear cells were collected from the peripheral blood of rats and cultured with 2% FBS EGM-2 for 21 days. The mononuclear cells presented a cobblestone appearance under a light microscope (**a**). The mononuclear cells were also stained with PE-labelled VEGFR2 (**b**), FITC-labelled CD34 (**c**), DiI-acetyl-labelled LDL (**d**) and FITC-UEA-1 (**e**) under a fluorescence microscope to identify the ECFCs (× 400 magnification). The cells were further analysed with a flow cytometer to confirm the ECFCs (**f**). The cells were stained with FITC-anti-CD14 and PE-anti-CD45 (**g**, **h**). The percentage of CD14-CD45- cells was approximately 99.8%

Cobble-shaped ECFCs were observed (Fig. 1a). The ECFCs exhibited positive fluorescence signals for VEGFR-2 and CD34 staining (Fig. 1b, c) and for DiI and UEA (Fig. 1d, e). To identify the sub-type of ECFCs, the cells were analysed for the expression of CD14 and CD45 using flow cytometry (Fig. 1f–h). All the cells were CD14^−^/CD45^−^ (Fig. 1g, h). These data indicated that the mononuclear cells were late outgrowth ECFCs [19].

### ECFC proliferation capacity and tube formation

**Fig. 2:**
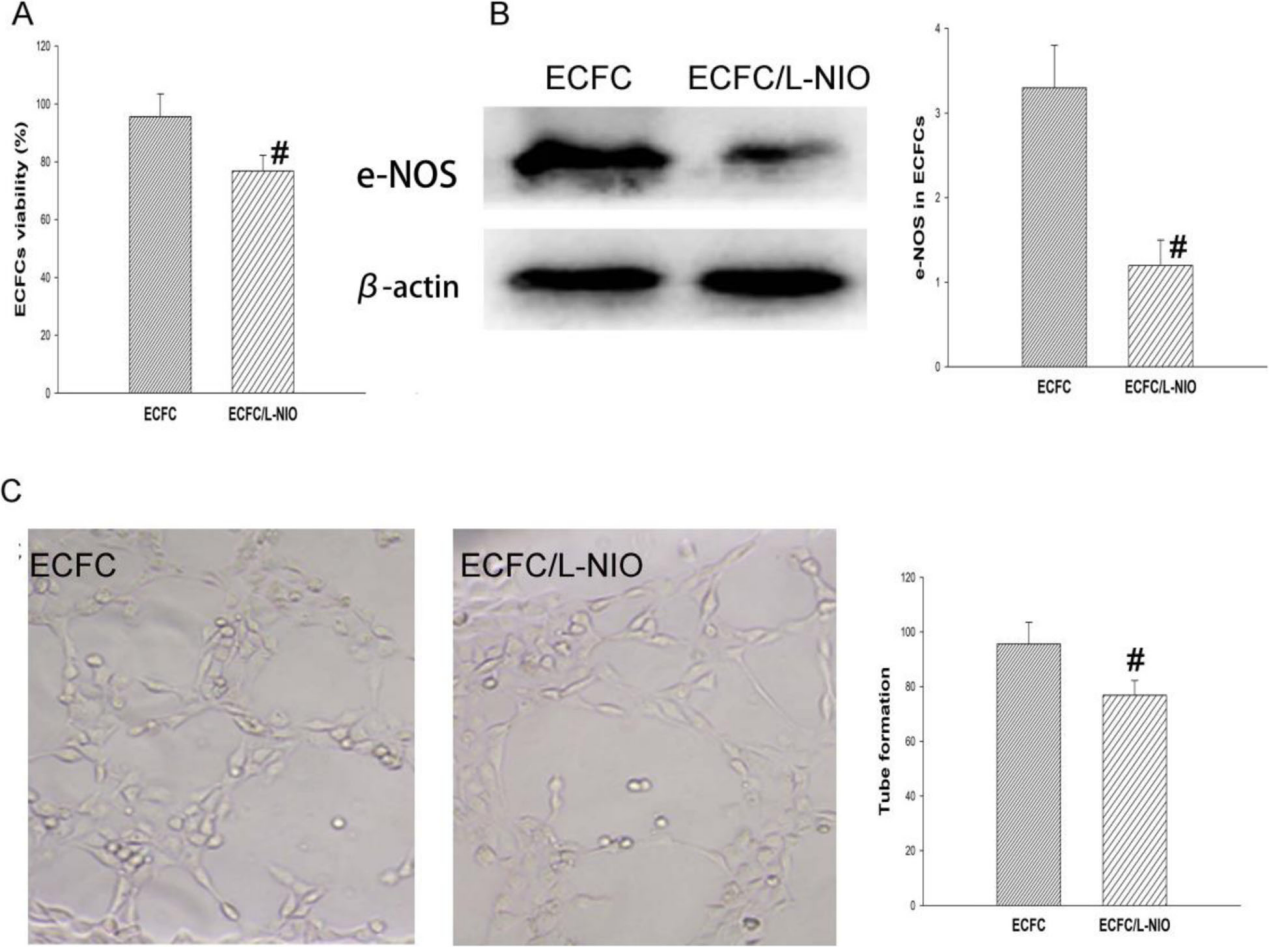
ECFC proliferation ability and tube formation. ECFCs treated with or without L-NIO were compared. The cellular viability and proliferation of ECFCs were judged by the MTT assay. Compared with that of the normal ECFCs, the viability of the ECFCs that received L-NIO treatment was significantly decreased (**a**). e-NOS protein expression was detected by Western blotting to investigate the effect of L-NIO on the expression of ECFCs. Compared with that of the ECFC group, e-NOS protein expression was reduced by L-NIO treatment. Representative images and quantitative analysis (**b**). Effect of L-NIO on the formation of ECFC tube-like structures. The results showed that compared with the cells of the normal ECFC group, ECFC cells pre-treated with L-NIO demonstrated a marked reduction in the number of tubes that were formed. Representative images and quantitative analysis (**c**) (**#**, *P*<0.05)

Compared with normal ECFCs, the viability of the ECFCs that received the L-NIO treatment was significantly decreased (95.6 ± 7.9 vs 80.0 ± 5.43) (*P* < 0.001). We also found that L-NIO significantly reduced the expression of eNOS in the ECFCs (4.3 ± 0.8 vs 1.2 ± 0.3) (*P* < 0.001) (Fig. 2). Compared with normal ECFCs, the tube formation capacity of ECFCs was significantly inhibited by the L-NIO (*P* < 0.001) (Fig. 2).

### Detection of ECFCs in the lung tissue

**Fig. 3:**
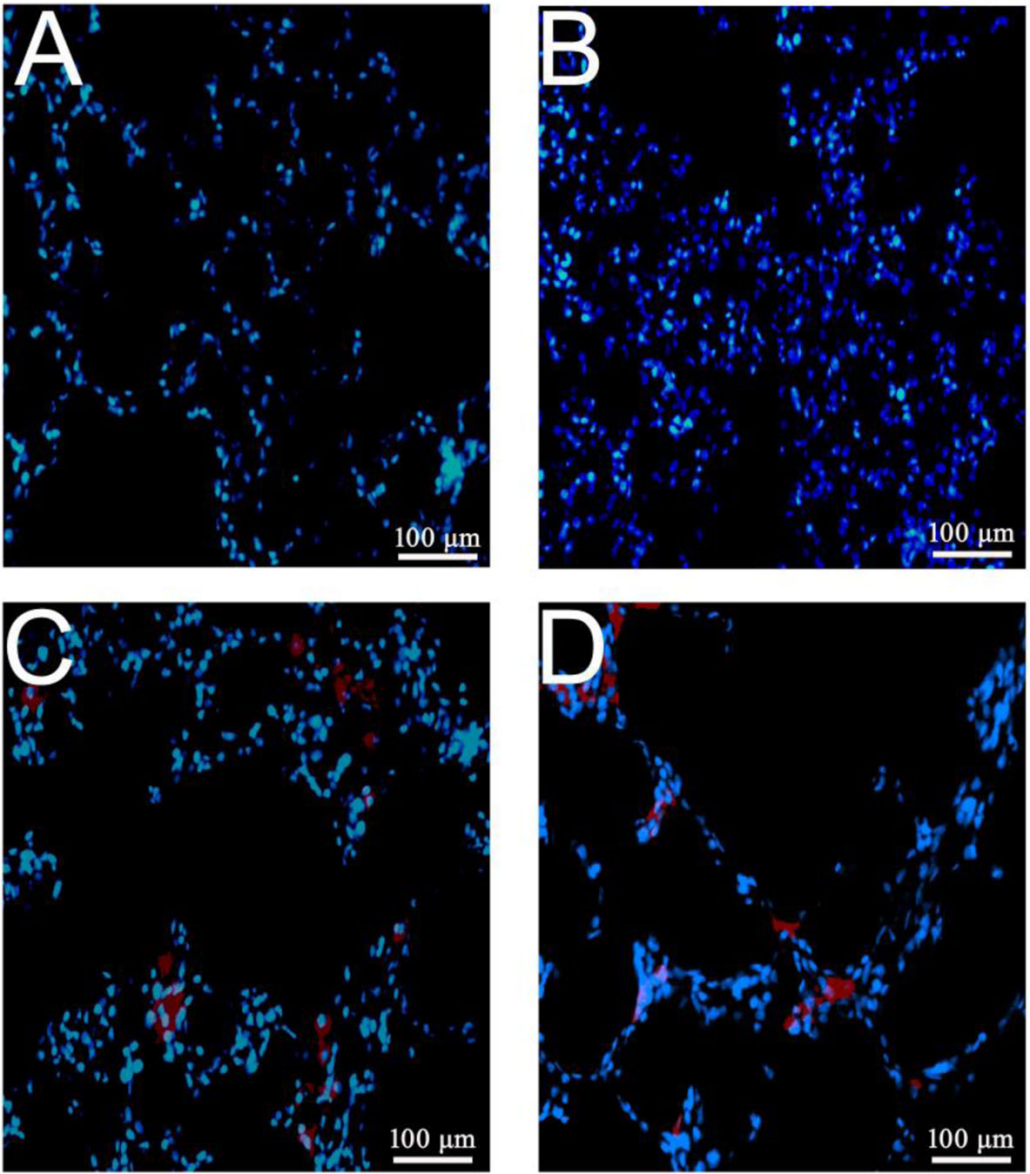
ECFC distribution in the lung tissue. Approximately 10^6^ cells/ml ECFCs were pre-labelled with DiI-acetyl-LDL for 2 h before injection. Twenty-four hours after CPB, the lung tissue sections (4 μm) were stained with DAPI. The ECFCs were examined with a fluorescence microscope. The results showed that there were no detectable ECFCs in rats from the sham group (**a**) or CPB group (**b**). More ECFCs were detected in the ECFC group (**c**) rats than in the ECFC/L group (**d**) rats. The infiltration of the ECFCs into the lung tissue was significantly reduced by the eNOS inhibitor

The rats in the ECFC and ECFC/L groups received an injection of ECFCs with or without L-NIO pre-treatment. No ECFCs were detected in the sham and CPB groups (Fig. 3a, b). The number of ECFCs in the lung tissue from rats in the ECFC and ECFC/L groups was calculated under a fluorescence microscope (Fig. 3c, d). The number of ECFCs in the ECFC/L group was significantly smaller than that in the ECFC group (7.3 ± 2.1% vs. 13.7 ± 3.5%, *P* < 0.05).

### ECFC reduced histological injury induced by CPB

**Fig. 4:**
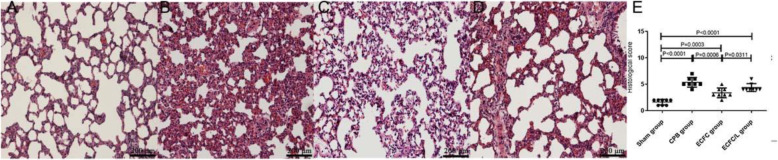
ECFCs attenuated lung damage after CPB. Assessment of lung histopathologic injury by HE staining. No histopathological changes were found in the sham group rats (**a**). After 24 h of CPB, many inflammatory cells infiltrated the lung tissue. Haemorrhage, oedema and broken alveoli were found in the CPB group (**b**). Compared with that in the CPB group, pathological injury was mitigated by the ECFCs (**c**), and the protective effect of ECFCs was reduced by the eNOS inhibitor (**d**). Quantitative analysis (**e**). (black circle, sham group; black square, CPB group; black up-pointing triangle, ECFC group; black down-pointing triangle, ECFC/L group)

Compared to the sham group, we found typical pathological changes in the CPB group, including lung oedema, bleeding, infiltration of inflammatory cells and damaged alveoli. Compared with that in the CPB group, the lung injury score was significantly reduced in the ECFC group. However, the protective effect of ECFCs on lung injury was reduced by the L-NIO (Fig. 4).

### ECFCs improved the alveolar-capillary permeability after CPB

**Fig. 5:**
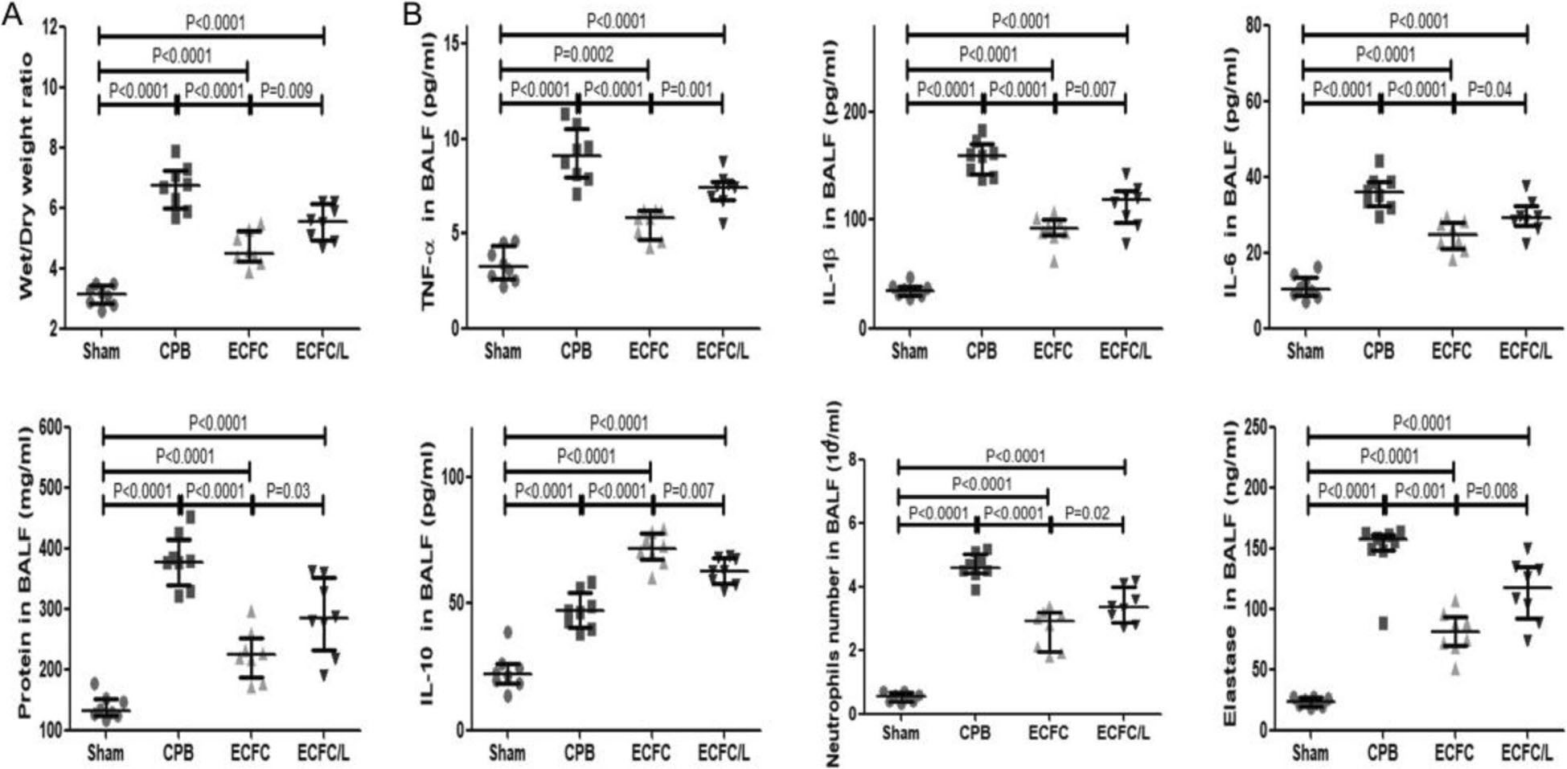
ECFCs improved capillary permeability and reduced local inflammation after CPB. After CPB for 24 h, the wet/dry ratio deteriorated. Compared with the wet/dry ratio in the CPB group, that in the ECFC group was decreased. The positive effect of ECFCs on the capillaries was reduced by L-NIO (**a**). After CPB, the cytokine levels, protein concentration and the number of cells in the BALF were significantly increased. Compared to the CPB treatment, ECFCs significantly reduced the concentrations of TNF-α, IL-1β and IL-6, the protein concentrations, the number of neutrophils and the level of neutrophil elastase in the BALF but the levels of IL-10 in the ECFC group were elevated. The regulatory effect of ECFCs on inflammatory factors and proteins was partly reversed by L-NIO (**b**). (black circle, sham group; black square, CPB group; black up-pointing triangle, ECFC group; black down-pointing triangle, ECFC/L group)

Compared with the sham group, PaO_2_/FiO_2_, the lung tissue W/D ratio and the concentration of protein in BALF were markedly deteriorated by CPB. After stimulation with CPB, PaO_2_/FiO_2_ was increased, but the protein levels and the W/D ratio were decreased by ECFCs compared with those of the CPB group. Compared with those in the ECFC group, the improvements in PaO_2_/FiO_2_, protein levels and W/D weight ratios were significantly mitigated in the ECFC/L group (Table 1 and Fig. 5a).

**Table 1:**
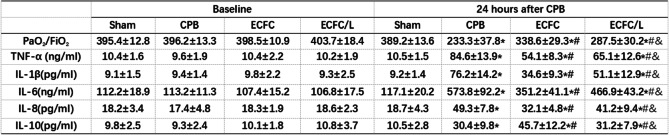
ECFCs improved capillary permeability and reduced inflammation after CPB

Data were collected at pre-CPB and 24 h after CPB. After CPB for 24 h, the PaO_2_/FiO_2_ deteriorated. Compared with the CPB group, in the ECFC group, the PaO_2_/FiO_2_ was increased. The positive effect of ECFCs on the capillaries was reduced by L-NIO. After CPB for 24 h, the systemic inflammation response was characterized by a significant increase in TNF-α, IL-1β, IL-6, IL-8 and IL-10. Compared to the CPB treatment, ECFCs significantly reduced the concentrations of TNF-α, IL-1β, IL-6 and IL-8 but elevated the levels of IL-10 in the ECFC group. The effect of ECFCs on systemic inflammation was reduced by L-NIO (the data are presented as the mean ± SD)

*P < 0.05 compared with the sham group

^#^*P* < 0.05 compared with the CPB group

^&^*P* < 0.05 compared with the ECFC group

### ECFCs inhibited local and systemic inflammation after CPB

**Fig. 6:**
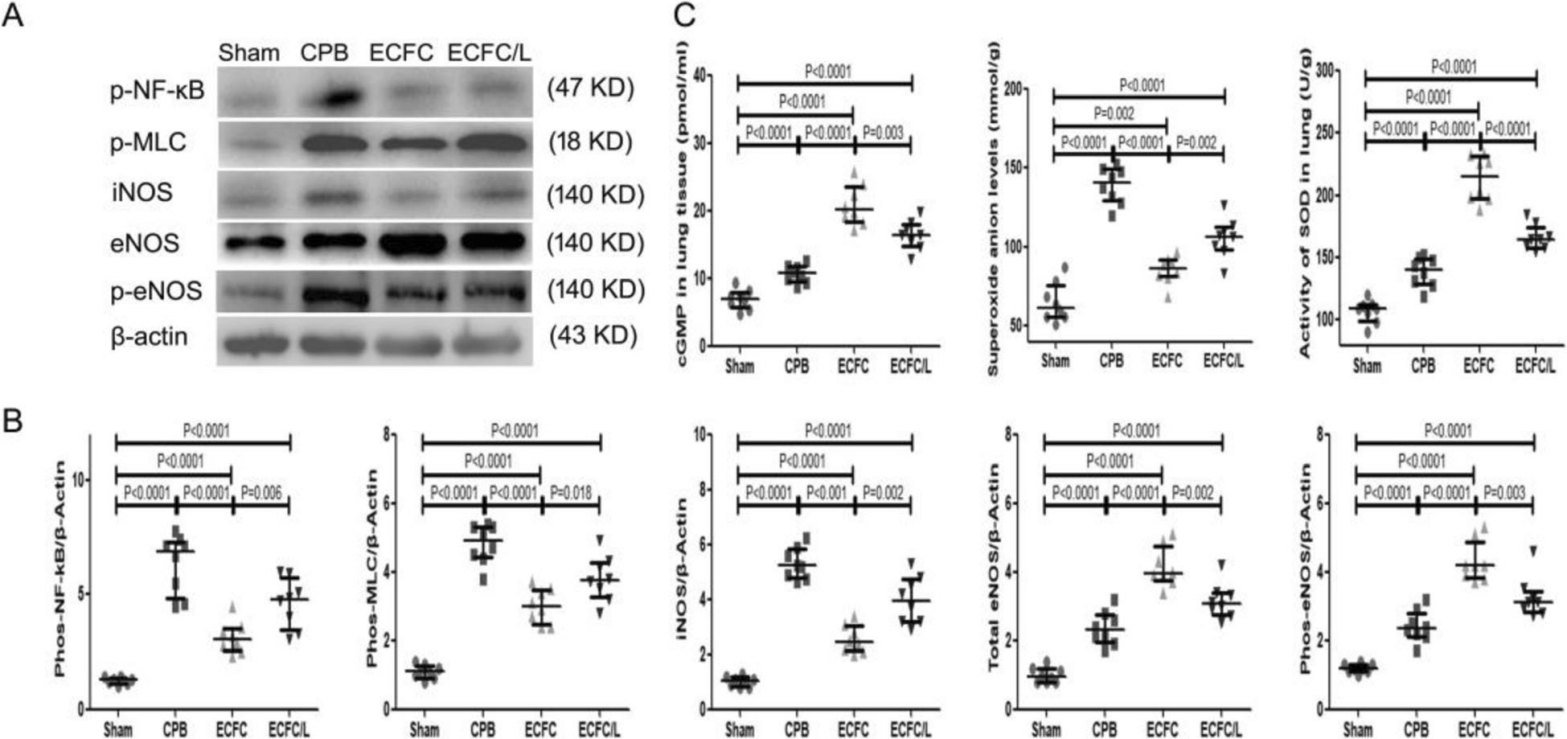
Effect of ECFCs on NF-kB and oxidative stress-related proteins. After CPB for 24 h, the expression of phosphorylated NF-κB and phosphorylated MLC in the lung tissue was significantly increased. These increases in protein levels were inhibited by ECFCs, and the effect of ECFCs was reduced by L-NIO (**a**). Quantitative analysis (**b**). After CPB, the levels of cGMP, superoxide anion, iNOS, eNOS and phosphorylated eNOS and the activity of SOD were increased. The increase in the oxidative stress response was inhibited by ECFCs in the ECFC group, and the inhibition of ECFCs on redox was reversed by L-NIO (A). Quantitative analysis (**c**). (black circle, sham group; black square, CPB group; black up-pointing triangle, ECFC group; black down-pointing triangle, ECFC/L group)

Compared with those in the sham group, the cytokine levels and the number of cells were significantly increased in rats that received CPB. Compared to the CPB group, the ECFC group exhibited significantly reduced concentrations of TNF-α, IL-1β and IL-6 but elevated levels of IL-10. The ECFCs also decreased the number of neutrophils and neutrophil elastase in the BALF (Fig. 5b). Moreover, the expression of phosphorylated NF-kB and MLC was also inhibited by ECFCs (Fig. 6a, b).

Second, pro-inflammatory factors in the serum were also reduced by ECFCs, but the anti-inflammatory factor IL-10 was upregulated by the ECFCs. Compared with the ECFC group, the regulatory effect of ECFCs on inflammatory factors and proteins was partly reversed by L-NIO (Table 1).

### ECFCs reduced the oxidative stress response

Compared with the sham group, the cGMP and superoxide anion levels and the SOD activity were increased after CPB. The increase in the oxidative stress response was inhibited by the ECFCs in the ECFC group, and the inhibition of ECFCs on redox was reversed by the L-NIO. Moreover, iNOS expression was also reduced by the ECFCs, and this reduction was lessened by the L-NIO. In contrast to iNOS, eNOS and phosphorylated eNOS were promoted by the ECFCs, and the promotion was weakened by the L-NIO (Fig. 6a, c).

### ECFCs attenuated apoptosis of the endothelium and epithelium after CPB

**Fig. 7:**
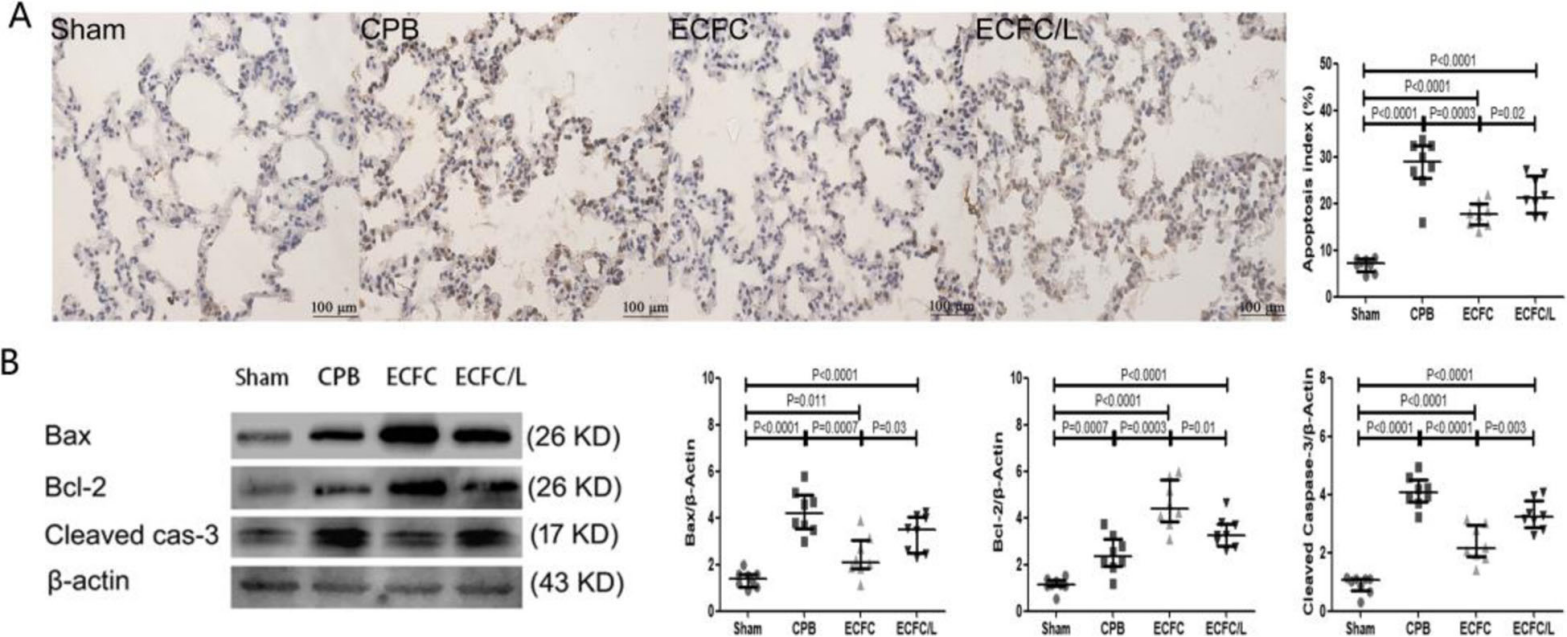
ECFCs reduced CPB-induced apoptosis. Cell apoptosis was determined by the TUNEL assay. Compared with the sham group, in the CPB group, many apoptotic cells were found, and cell apoptosis was reduced by ECFCs. The anti-apoptotic effect of ECFCs was reversed by L-NIO. Representative images and quantitative analysis (**a**). After CPB for 24 h, the levels of Bax, BcL-2 and cleaved caspase 3 relative to that of β-actin in the lung tissues were upregulated by CPB. Compared with those in the CPB group, Bax and cleaved caspase-3 levels were reduced and Bcl-2 was promoted by ECFCs. The regulatory effect of ECFCs was reversed by L-NIO. Representative images and quantitative analysis (**b**). (black circle, sham group; black square, CPB group; black up-pointing triangle, ECFC group; black down-pointing triangle, ECFC/L group)

In the sham group, few apoptotic cells were detected. After CPB, many apoptotic endothelial and epithelial cells were observed in the lung tissue. Compared with that in the CPB group, the number of apoptotic cells was significantly reduced in the ECFC group. However, the number of apoptotic cells in the ECFC/L group was significantly increased compared with that in the ECFC group (Fig. 7a). We also found that the Bax, Bcl-2 and cleaved caspase-3 levels were significantly increased in the rats that received CPB compared with those in the sham group. Compared to those in the CPB group, Bax and cleaved caspase-3 levels were downregulated, but Bcl-2 was upregulated by the ECFCs. Compared with that in the ECFC group, the regulatory effect of ECFCs on apoptosis was reduced by L-NIO (Fig. 7b).

## Discussion

In this study, the ECFCs ameliorated lung injury, improved alveolar-capillary permeability and gas exchange function, attenuated the redox, reduced local and systemic inflammation and inhibited apoptosis induced by CPB.

During CPB, lung ischaemia and the introduction of an artificial circuit into the blood resulted in severe local and systemic inflammation [20], which led to prolonged mechanical ventilation, a prolonged stay in the ICU and even respiratory failure and increased mortality. ECFCs can reduce the ventilator-induced lung injury in rats with ARDS and protect against renal reperfusion injury via anti-inflammatory effects [10, 11]. Acute lung injury after CPB is the major risk factor against patient recovery. Furthermore, we did not find serious clinical long-term pulmonary complications after CPB [21]. Therefore, in this study, we administered intravenous ECFCs to observe the effect of ECFCs on acute lung injury after CPB. Moreover, to avoid the effect of haemodilution and the pressure of the roller pump on ECFCs, we injected the ECFCs after withdrawal of CPB.

In this study, we found that the ECFCs significantly improved gas exchange function and mitigated histological changes [22]. These results indicated that ECFCs can ameliorate lung injury. As previous studies indicated, the lung injury after CPB was associated with inflammation, oxidative stress response and apoptosis. In this study, we investigated the mechanism of ECFCs in lung injury after CPB based on inflammation, the oxidative response and apoptosis.

Many studies have suggested that the imbalance of inflammation plays a key role in the pathogenesis of lung injury after CPB [3]. During CPB, activation of NF-kB promoted the release of chemokines, such as MCP-1 and ICAM-1, and further recruited the neutrophils that migrated into the lung tissue [23]. The activated inflammatory cells released TNF-α, IL-1β, IL-8, elastase and MMP-9, which aggravated local inflammation and the systemic inflammatory response [24]. In this study, the results indicated that ECFCs mitigated inflammation after CPB, which was consistent with previous studies [14]. This anti-inflammatory effect of ECFCs may be associated with inhibition of chemokines (MCP-1 and ICAM-1) [11] and regulation of the immune response [25]. The regulation of ECFCs on inflammation was mainly attributed to the inhibition of NF-kB [26]. Moreover, the capacity of ECFCs to induce anti-inflammatory IL-10 also played an important role. IL-10 opposed the injurious effect of TNF-α, IL-1β and IL-6 and reduced the recruitment of inflammatory cells [27]. The protective effects of ECFC on lung injury also depended on the regulatory effect of ECFC on MLC [28]. During inflammation, MLC was activated and phosphorylated after endothelial injury [29] and further damaged the contractile elements of the endothelium [30]. In this study, ECFC treatment improved the pulmonary endothelial barrier and ameliorated pulmonary oedema, and this result was consistent with our previous study [14].

Under normal conditions, the bronchial arteries provide only 3–5% of the total blood volume to the lungs [31], which significantly declines [4] following CPB onset, leading to lung ischaemia/reperfusion injury [4]. During CPB, the injured endothelial cells can release iNOS induced by pro-inflammatory factors [32], which then produces NO and reactive nitric species [33], further contributing to endothelium injury. In contrast to iNOS, the eNOS immunoreactivity increased significantly [34], and the NO released from eNOS controls the vasodilation, vascular remodelling and angiogenesis [35]. The eNOS was regarded as a protective factor for endothelial cells [36]. The NO released from eNOS not only protected the endothelial cells but also stimulated the cGMP. As the second messenger of NO, the NO-cGMP signalling pathway played a key role in vascular tone and protected against lung injury [36–38]. The cGMP can block ROS formation or enhance ROS scavenging by a protein kinase G-dependent mechanism [39]. Moreover, during CPB, the superoxide anion will be released from the activated neutrophils and directly damage the lung tissue. To compensate for the oxidative stress response, SOD will be activated and will inhibit the production of superoxide anion. In this study, we found that ECFCs significantly reduced the expression of iNOS and superoxide anion but promoted the expression of eNOS and phosphorylated eNOS. The ECFCs also increased the levels of cGMP and the SOD activity. These results suggested that ECFCs could reduce the lung injury by inhibiting the oxidative stress response.

After CPB, apoptosis also played a pivotal role in lung injury [40]. Both inflammatory factors (TNF-α) and reactive oxygen species induced by lung ischaemia lead to cell apoptosis [41]. The apoptotic endothelium and epithelium deteriorated pulmonary function and increased capillary permeability. In this study, the ECFCs significantly reduced cell apoptosis after CPB. During apoptosis, Bax and Bcl-2 play a pivotal role. Bax is a pro-apoptosis protein that sends the apoptosis signal and promotes the activation of caspase-3 to produce cleaved caspase-3, which cuts the DNA and results in cell apoptosis. In contrast to Bax, Bcl-2 is an anti-apoptosis protein and inhibits the role of Bax. The ratio of Bax to Bcl-2 usually determines the survival or apoptosis of cells [42]. In this study, the ECFCs significantly reduced the expression of Bax and cleaved caspase-3 but increased the expression of Bcl-2. Moreover, the capacity of ECFCs to reduce apoptosis was also attributed to the capacity of ECFCs to decrease TNF-α expression. The inhibitory effect of ECFCs on apoptosis also contributed to the protective effect of ECFCs against lung injury [43].

eNOS has been suggested to play a key role in the bioactivation of early endothelial progenitor cells [12]. In our previous study, we also demonstrated that the eNOS inhibitor significantly decreased the infiltration of endothelial progenitor cells in transplanted lung tissue by interfering with eNOS expression [14]. In this study, we administered the NOS inhibitor L-NIO to ECFCs to test whether eNOS plays a crucial role in late endothelial progenitor cells. In the in vitro study, the L-NIO significantly reduced the viability and tube formation activity of ECFC. In the in vivo study, the L-NIO notably decreased the number of ECFCs in the lung tissue and weakened the protection of ECFCs on lung injury induced by CPB. This result was consistent with our previous studies.

### Limitation

There are some limitations in this study. The first is that there is no uniform consensus regarding unique and specific markers of ECFCs [44], although some articles suggested that the method used in this study can identify ECFCs [19]. Specific markers of ECFCs are still needed. The second is that the ECFCs were harvested after 21 days of culture. This duration is too long for applications in some patients receiving CPB. However, in clinical work, there are several patients with severe preoperative organ dysfunction who need long-term treatments. Therefore, ECFCs may benefit these patients. Moreover, the effects of ECFCs in rat studies may not be analogous to those in humans after CPB. In this study, the rats that received the CPB were young, but CPB is usually applied in the elderly in clinical work. We will administer the ECFC in older rats to estimate the benefits of ECFC on CPB. In addition, we did not monitor the pulmonary physical function after CPB because we did not have a professional monitor. Moreover, although we found that eNOS played an important role, the mechanism by which ECFCs function is still unclear. Considering the regulated effect of PI3K/AKT1 on eNOS, we will prepare an shRNA to interfere with PI3K/AKT1 expression to further explore the possible mechanism of ECFCs on lung injury in our future study.

## Conclusion

The results of this study suggested that ECFCs can attenuate lung injury, improve endothelial structure and function, reduce local and systemic inflammation and inhibit cell apoptosis. The protective effect of ECFCs on lung injury after CPB is mainly associated with eNOS in ECFCs.

## Data Availability

All data generated or analysed during this study are included in this published article.

## Abbreviations

CPB: Cardiopulmonary bypass
ECFCs: Endothelial colony-forming cells
BALF: Bronchoalveolar lavage fluid
UEA-1: *Ulex europaeus* agglutinin-1
LDL: Llow-density lipoprotein
VEGFR: Vascular endothelial growth factor receptor
L-NIO: N5-(1-iminoethyl)-l-ornithine
MTT: 3-(4,5-dimethylthiazol-2-yl)-2,5-diphenyltetrazolium bromide
Vt: Tidal volume
RR: Respiratory rate
FiO2: Fraction of inspired oxygen
PEEP: Positive end-expiratory pressure
W/D: Wet/dry weight
DAPI: 4,6-Diamidino-2-phenylindole
MCP-1: Monocyte chemotactic protein 1
ICAM-1: Intercellular cell adhesion molecule-1
MLC: Myosin light chain
