# Correction to: Endothelial colony-forming cells reduced the lung injury induced by cardiopulmonary bypass in rats

**DOI:** 10.1186/s13287-021-02567-4

**Published:** 2021-09-03

**Authors:** Haibin Sun, Xiaoqing Zhao, Qihang Tai, Guangxiao Xu, Yingnan Ju, Wei Gao

**Affiliations:** 1 Department of Anesthesiology, The Second Affiliated Hospital of Harbin, Medical University, Harbin, China; 2 Department of ICU, Tumor Hospital of Harbin Medical University, Harbin, China

## Correction to: Sun et al. Stem Cell Research & Therapy (2020) 11:246 https://doi.org/10.1186/s13287-020-01722-7

Following publication of the original article [1], the authors have identified that the incorrect images were included for Fig. 4B due to an error with the image selection during manuscript preparation. Moreover, the data presentation in Fig. 4E should be presented with mean and SD.

Figure 4 should therefore be updated as follows:

**Fig. 4:**
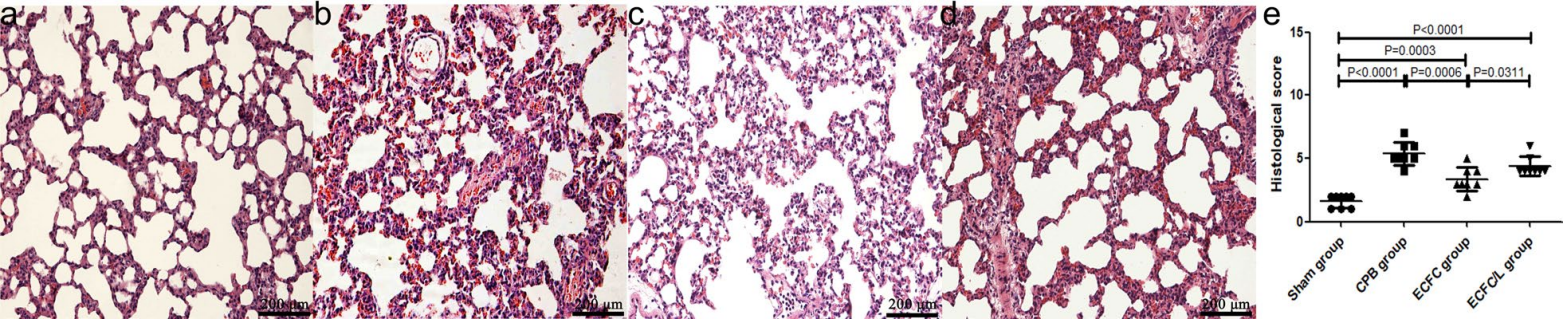
ECFCs attenuated lung damage after CPB. Assessment of lung histopathologic injury by HE staining. No histopathological changes were found in the sham group rats (**a**). After 24 h of CPB, many inflammatory cells infiltrated the lung tissue. Haemorrhage, oedema and broken alveoli were found in the CPB group (**b**). Compared with that in the CPB group, pathological injury was mitigated by the ECFCs (**c**), and the protective effect of ECFCs was reduced by the eNOS inhibitor (**d**). Quantitative analysis (**e**). (black circle, sham group; black square, CPB group; black uppointing triangle, ECFC group; black down-pointing triangle, ECFC/L group)
